# Comparison of stereotactic body radiation therapy with hepatic resection and radiofrequency ablation as initial treatment in patients with early-stage hepatocellular carcinoma

**DOI:** 10.3389/fonc.2022.948866

**Published:** 2022-11-21

**Authors:** Zi-liang Yang, Xu-qi Sun, Yu-hao Tang, Pei-yao Xiong, Li Xu

**Affiliations:** ^1^ Department of Liver Surgery, Sun Yat-sen University Cancer Center, Guangzhou, Guangdong, China; ^2^ State Key Laboratory of Oncology in South China, Guangzhou, Guangdong, China; ^3^ Collaborative Innovation Center for Cancer Medicine, Guangzhou, Guangdong, China; ^4^ Department of Radiotherapy, The First Affiliated Hospital of Sun Yat-sen University, Guangzhou, Guangdong, China

**Keywords:** stereotactic body radiation therapy, liver resection, radiofrequency ablation, hepatocellular carcinoma, overall survival

## Abstract

**Background:**

Stereotactic body radiation therapy (SBRT) has emerged as a novel intervention for early-stage hepatocellular carcinoma (HCC). The outcomes of SBRT, liver resection (LR), and radiofrequency ablation (RFA) as the initial treatment for AJCC stage I HCC patients remain unclear.

**Methods:**

Patients with AJCC stage I HCC from the Surveillance, Epidemiology and End Results database were analyzed for survival rates using the Kaplan–Meier method and stratified according to tumor size: S subgroup (≤2 cm), M subgroup (>2–3 cm), and L subgroup (>3 cm). For factors including age, year of diagnosis, sex, race, grade, tumor size, AFP, and fibrosis score, propensity score matching was performed to eliminate the imbalance of baseline features and selection bias during groups.

**Results:**

A total of 4,002 patients were included; the difference in median overall survival (mOS) between the SBRT group and the LR or RFA group in the S subgroup was statistically insignificant (*p*=0.109 and *p*=0.744), while that of the RFA group was significantly worse than that of the LR group (*p <*0.001). In the M and L subgroups, the mOS of the SBRT group was worse than that of the RFA group (*p*=0.040 and *p*<0.001, respectively). The mOS of LR was the best when compared with either the SBRT or RFA group regardless of the subgroup M or L (all *p*<0.001).

**Conclusion:**

For HCC ≤ 2 cm, SBRT can be used as an alternative treatment for RFA. For patients with HCC larger than 2 cm, RFA can provide better long-term survival than SBRT, while LR remains the best choice.

## Introduction

Hepatocellular carcinoma (HCC) is the sixth most common diagnosed malignancy worldwide with increasing incidence and mortality (1). For a majority of patients, the disease tended to have already reached advanced stage when first diagnosed and thus unresectable, taking chemotherapy, target therapy, and immune checkpoint blockade (ICI) as first- or later-line treatment. However, more and more patients diagnosed with an early-stage HCC profited by the spread of health screening. For early-stage HCC, liver transplantation can confer excellent survival to patients who fulfill the Milan criteria; yet, it is not feasible for the majority in China due to donor shortage and high medical cost (2). Liver resection (LR) is adopted as the first-line therapy for early-stage HCC with favorable 5-year survival of 60%–80%; however, some patients are not candidates for various reasons such as insufficient remnant liver and anatomical relations (3, 4). Radiofrequency ablation (RFA) is a minimally invasive intervention, of which the local control rates (LCRs) can reach 70%–90%; thus, it is recommended as the first-line therapy for inoperable patients with small HCC (3, 5). Whether RFA can achieve comparable overall survival (OS) to LR still remains controversial (6). There also exist some limitations in RFA including heat sink and unstable efficacy affected by tumor location or size (7). Stereotactic body radiation therapy (SBRT) is the standard intervention for inoperable stage I non-small-cell lung cancer, featured by the delivery of high dose of radiation in a highly targeted fashion. In recent years, SBRT has emerged as an alternative local treatment modality to RFA in HCC (8, 9).

Studies have reported that SBRT has similar LCRs compared with RFA, and its efficacy is not limited by tumor size (9, 10). Yet, most retrospective studies are single institutional and affected by relatively small sample size. Even results from multi-center studies show great variations, while prospective randomized controlled trials (RCTs) directly comparing the efficiency of SBRT and RFA are still pending. Wahl et al. reported that SBRT provided comparable survival to RFA for inoperable HCC, while another study by Rajyaguru et al. showed that RFA is associated with superior survival than SBRT in non-surgically patients (11, 12). Varied cutoffs of tumor size were selected for subgroup analysis for survival benefits of SBRT in HCC, including 2 or 3 cm (11, 13). Moreover, unlike studies comparing SBRT with RFA, few studies focused on the comparison of SBRT with LR. Su et al. reported that SBRT has similar LCRs with LR for small HCC, but the sample size of this single-institutional research was quite small (n = 35 for the surgery group) (14). In the setting of absence of RCTs, an observational study from a large-scale population could improve the level of evidence for the comparison of SBRT with LR and RFA. To figure out the optimal initial treatment for adult patients with early HCC, we conducted the present study based on interventions and tumor size by using the data from the Surveillance, Epidemiology, and End Results (SEER) database.

## Materials and methods

### Patients and experimental design

We collected data from the SEER database, which consists of 17 population-based cancer registries, covering approximately 30% of the total population in the United States (US) (15), and specified a retrospective dataset between January 2010 and December 2015 to the best balancing of enough follow-up time and the mature treating technology. The entire study population enrolled patients with the International Classification of Diseases for Oncology codes, namely, 8170/3, 8171/3, 8172/3, 8173/3, 8174/3, and 8175/3, and the site code, C22.0. Patients with American Joint Committee on Cancer (AJCC) stage I HCC who received SBRT, RFA, or LR as initial treatments were eligible for this study. Patients whose tumors were concomitant with other tumors or lack follow-up information were excluded. Information of this dataset was extracted using SEER*Stat software version 8.3.5.

Patients were divided according to their initial treatment as SBRT group, RFA group, and LR group. The SRBT group consisted of patients who did not receive any surgery (Surg Prim Site code: 0) and with beam radiation in the radiation code, which excluded those performing SBRT as adjuvant or neoadjuvant therapies. The RFA group consisted of patients with surgery code of only 16. The LR group included patients who received resection with surgery codes 20–26, 30, 36–38, 50–52, 59, and 60. Patients were then stratified in subgroups according to their largest tumor size: S subgroup (≤2 cm), M subgroup (>2–3 cm), and L subgroup (>3 cm). The main study endpoint is overall survival (OS) defined as the time from the date of initial treatment to death or last follow-up.

### Statistical analysis

The comparisons of clinical features at baseline among different treatment groups were performed with chi-squared tests. The stratified analyses for survival rates according to treatments in different subgroups were performed with Kaplan–Meier (K-M) method. Comparisons between groups were analyzed with log-rank test. Cox proportional hazards regression model was adopted to calculate hazard ratios in terms of treatments for groups with different tumor sizes. Propensity score matching (PSM) was performed to eliminate the imbalance of baseline features and selection bias among groups. PSM was performed with regard to factors including age, year of diagnosis, sex, race, grade, tumor size, AFP, and fibrosis score characteristics, with a caliper value of 0.02 and at a ratio of 1:1 between RFA group and LR group while a ratio of 1:3 between SBRT and RFA or LR. The SEER database records AFP and fibrosis score as discontinuous variables (AFP: code 20 for within normal limit, code 10 for elevated ones; fibrosis score: score of 0–4 for non-moderate ones, score of 5–6 for severe cirrhosis ones), so these two variables were included as categorical variables in multivariate Cox regression model. A two-tailed *p*-value <0.05 was considered to be statistically significant. All data analyses were performed with IBM SPSS 24.0 and R version 3.2.2.

## Results

### Baseline and clinical features

**Figure 1:**
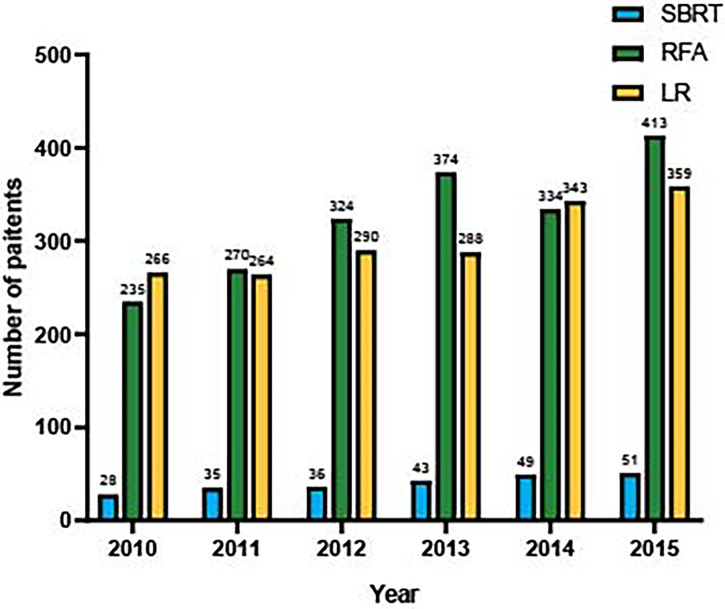
Number of eligible patients underwent SBRT, RFA and LF in SEER database, from 2010 to 2015.

This study included 4,002 patients diagnosed with AJCC stage I HCC in the SEER database between January 2010 and December 2015. The baseline demographic and clinical characteristics of patients are presented in **Table 1**. The median age of the whole study population was 64 years (range, 19–99 years). SBRT, RFA, and LR were performed in 242 (6.1%), 1,950 (48.7%), and 1,810 (45.2%) patients, respectively. Moderately to well-differentiated HCC ranked the most common pathological type in all groups. The SBRT group had a larger proportion of patients older than 60 years (67.4% *vs*. 60.4% or 64.2%). The RFA group had more patients with elevated AFP and high fibrosis scores compared with the other two groups. LR was adopted as the most common treatment for patients with HCC lesions measuring >2 cm (87.3% *vs*. 82.3% or 67.9%). For HCCs smaller than 3 cm, RFA was the most common intervention (70.2% *vs* 45.3% or 33.2%). The numbers of the three interventions performed during each year are shown in **Figure 1**. It was observed that there was an apparent elevation of included patients in the database over time for all interventions. Among them, SBRT had the least adoption yet the top growth rate, indicating an increasing attention by clinicians. RFA was quantitively the most popular intervention, showing a faster spread than LR.

**Table 1:** Baseline characteristics of the study population.

Variables	Total (n = 4002)	SBRT (n = 242)	RFA (n = 1950)	LR (n = 1810)	P value
Age, years					< 0.001
Median (IQR)	64 (58, 71)	67 (58,76)	63 (58, 70)	64 (58, 71)	
≤ 60	1499 (37.4%)	79 (32.6%)	772 (39.5%)	648 (35.8%)	
60-75	1935 (48.3%)	97 (40.0%)	927 (47.5%)	911 (50.3%)	
> 75	568 (14.6%)	66 (27.4%)	251 (13.0%)	251 (13.9%)	
Race					< 0.001
White	2528 (63.1%)	188 (77.6%)	1304 (66.8%)	1306 (57.2%)	
Black	469 (11.7%)	26 (10.7%)	217 (11.1%)	226 (12.4%)	
Other/Unknown	1005 (25.2%)	28 (11.7%)	429 (2.1%)	548 (30.4%)	
Gender					0.710
Male	2878 (71.9%)	172 (71.0%)	1414 (72.5%)	1292 (71.3%)	
Female	1124 (28.1%)	70 (29.0%)	536 (27.5%)	518 (28.7%)	
Tumor differentiation					< 0.001
Well	750 (18.7%)	37 (15.4%)	274 (14.0%)	439 (24.2%)	
Moderate	1257 (31.4%)	26 (10.7%)	335 (17.4%)	896 (49.5%)	
Poor	401 (10.2%)	16 (6.6%)	76 (3.8%)	309 (17.0%)	
Unknown	1594 (39.7%)	164 (67.3%)	1265 (64.8%)	166 (9.3%)	
AFP					< 0.001
Normal	1347 (33.6%)	87 (35.9%)	620 (31.7%)	640 (35.3%)	
Elevated	1832 (45.7%)	110 (45.6%)	988 (50.6%)	734 (40.5%)	
Unknown	832 (20.7%)	45 (18.5%)	342 (17.7%)	436 (24.2%)	
Fibrosis score					< 0.001
0-4	486 (12.3%)	7 (2.8%)	104 (5.3%)	375 (20.7%)	
5-6	1020 (25.4%)	46 (19.0%)	669 (34.3%)	305 (16.8%)	
Unknown	2496 (62.3%)	189 (78.2%)	1177 (60.4%)	1130 (62.5%)	
Tumor size, cm					< 0.001
≤ 2	900 (22.4%)	43 (17.7%)	626 (32.1%)	231 (12.7%)	
>2-3	1183 (29.5%)	67 (27.6%)	744 (38.1%)	372 (20.5%)	
> 3	1919 (48.1%)	132 (54.7%)	580 (29.8%)	1207 (66.8%)	

AFP, alpha fetoprotein.

### Overall survival and stratified analyses

**Figure 2:**
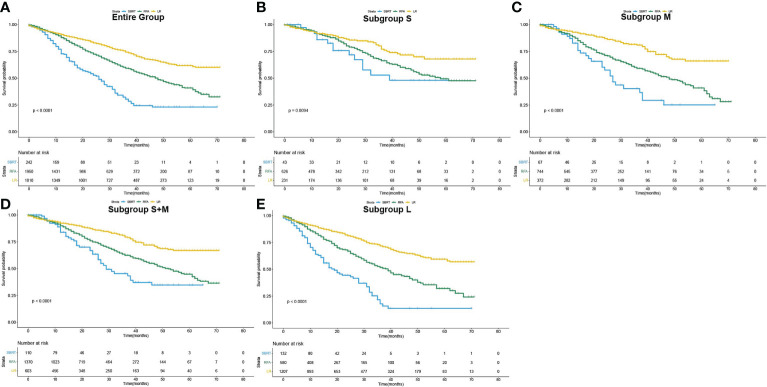
Overall survival curves of patients who underwent SBRT, RFA and LF as initial treatment (Kaplan–Meier method). **(A)** for the entire population, **(B–E)** for subgroup S, M, S+M and L, respectively.

The median overall survival (mOS) was 57 months for the entire group. During the follow-up, 1,142 (28.5%) deaths were observed. According to the K-M survival curves (**Figure 2A**), patients in the LR group showed the best OS with unreached mOS, followed by the RFA group with an mOS of 48 months, and the mOS of SBRT group was only 26 months. The survival difference among these three groups was significant (*p*<0.001). To further evaluate the outcomes among the three interventions, stratified analyses were conducted based on age at diagnosis, race, sex, year of diagnosis, tumor stage, AFP level, fibrosis score, and tumor size. The 3- and 5-year survival rates of patients in each subgroup are presented in **Table 2**. Obviously, patients receiving LR had better survival than those with RFA or SBRT in all subgroups.

**Table 2:** Results of stratified analyses for survival rates.

Variables	SBRT		RFA		LR		P value
	3-year (%)	5-year (%)	3-year (%)	5-year (%)	3-year (%)	5-year (%)	
Age, years							
≤ 60	36.9	34.0	61.9	45.8	76.6	65.5	< 0.001
60-75	31.5	21.8	57.0	40.2	75.6	62.1	< 0.001
> 75	20.8	11.1	58.0	26.4	63.7	51.4	< 0.001
Race							
White	33.9	25.6	55.5	37.6	73.1	60.9	< 0.001
Black	22.5	15.0	60.4	40.0	69.3	56.1	< 0.001
Other/Unknown	14.8	14.8	69.8	51.5	78.5	66.1	< 0.001
Gender							
Male	31.9	24.8	59.4	41.5	73.0	59.9	< 0.001
Female	26.8	19.6	58.8	39.0	77.6	67.5	< 0.001
Tumor differentiation							
Well	30.9	0	66.4	42.3	76.7	64.8	< 0.001
Moderate	0	0	59.6	39.4	77.2	65.4	< 0.001
Poor	23.4	23.4	46.5	33.6	64.2	50.1	< 0.001
Unknown	34.5	29.1	58.1	41.7	70.7	56.8	< 0.001
AFP							
Normal	28.3	24.7	66.0	44.9	81.4	68.9	< 0.001
Elevated	36.7	29.0	56.7	39.0	67.9	55.2	< 0.001
Unknown	16.7	0	53.9	40.1	74.8	63.5	< 0.001
Fibrosis score							
0-4	80.0	80.0	63.3	28.0	80.4	72.2	< 0.001
5-6	28.7	11.5	62.7	45.4	68.6	56.0	< 0.001
Unknown	30.0	24.7	56.6	39.7	73.7	59.8	< 0.001
Tumor size, cm							
≤ 2	52.9	48.1	66.3	49.4	77.1	67.9	0.009
>2-3	40.3	25.1	59.2	41.0	80.9	66.1	< 0.001
≤ 3	24.9	18.2	53.5	33.7	76.1	62.6	< 0.001
> 3	7.2	7.2	43.3	23.8	68.0	56.6	< 0.001

AFP: alpha fetoprotein.

The influence of three treatments on OS in subgroups of different tumor sizes was studied in detail. K-M analyses were performed based on interventions within each subgroup. The survival curves are shown in **Figures 2B–E**. For the S group, the difference in mOS between the SBRT group and the RFA group was not statistically significant (SBRT, 39 *vs*. 58 months, *p*=0.20), but the mOS of the SBRT and RFA groups were statistically worse than that of the LR group (SBRT, 39 months *vs*. LR unreached, *p*=0.017; RFA, 58 months *vs*. LR unreached, *p*=0.007). In the patients with tumors larger than 2 cm (subgroups M and L), the mOS of those who took SBRT as the initial treatment was worse than that of RFA (subgroup M: 27 *vs*. 48 months, *p*=0.006; subgroup L: 19 *vs*. 39 months, *p*<0.001). Among HCC patients with tumors larger than 2 cm (subgroups M and L), the median OS of LR patients was the best. A detailed median OS of patients in each treatment group and their comparison are shown in **Table 3**.

**Table 3:** Median survival time and hazard ratio of different groups before PSM.

Groups	Median overall survival (months)	95% CI	P value	Hazard Ratio	95% CI	P value
Entire Cohort (n=4002)	57.0	52.25-61.75				
SBRT (n=242)	26.0	22.07-29.93	< 0.001	1		–
RFA (n=1950)	48.0	43.91-52.09	< 0.001	0.492	0.41-0.60	< 0.001
LR (n=1810)	–	–	< 0.001	0.291	0.24-0.36	< 0.001
≤ 2 cm (n=900)	–	–				
SBRT (n=43)	39.0	–	0.297	1		–
RFA (n=626)	58.0	–	0.017	0.752	0.44-1.30	0.306
LR (n=231)	–	–	0.007	0.470	0.26-0.87	0.015
>2-3 cm (n=1183)	54.0	47.48-60.52				
SBRT (n=67)	27.0	22.53-31.47	0.006	1		–
RFA (n=744)	48.0	42.02-53.98	< 0.001	0.595	0.41-0.88	0.008
LR (n=372)	–	–	< 0.001	0.273	0.18-0.42	< 0.001
≤ 3 cm (n=2083)	61.0	–				
SBRT (n=110)	29.0	20.06-37.95	0.004	1		–
RFA (n=1370)	52.0	46.62-57.38	< 0.001	0.641	0.47-0.88	0.006
LR (n=603)	–	–	< 0.001	0.338	0.24-0.48	< 0.001
> 3 cm (n=1919)	55.0	47.93-62.07				
SBRT (n=132)	19.0	13.15-24.85	< 0.001	1		–
RFA (n=580)	39.0	33.41-44.57	< 0.001	0.475	0.37-0.62	< 0.001
LR (n=1207)	–	–	< 0.001	0.243	0.19-0.31	< 0.001

SBRT, stereotactic body radiation; RFA, radiofrequency ablation; LR, Liver resection;

CI, confidence interval.

**Figure 3:**
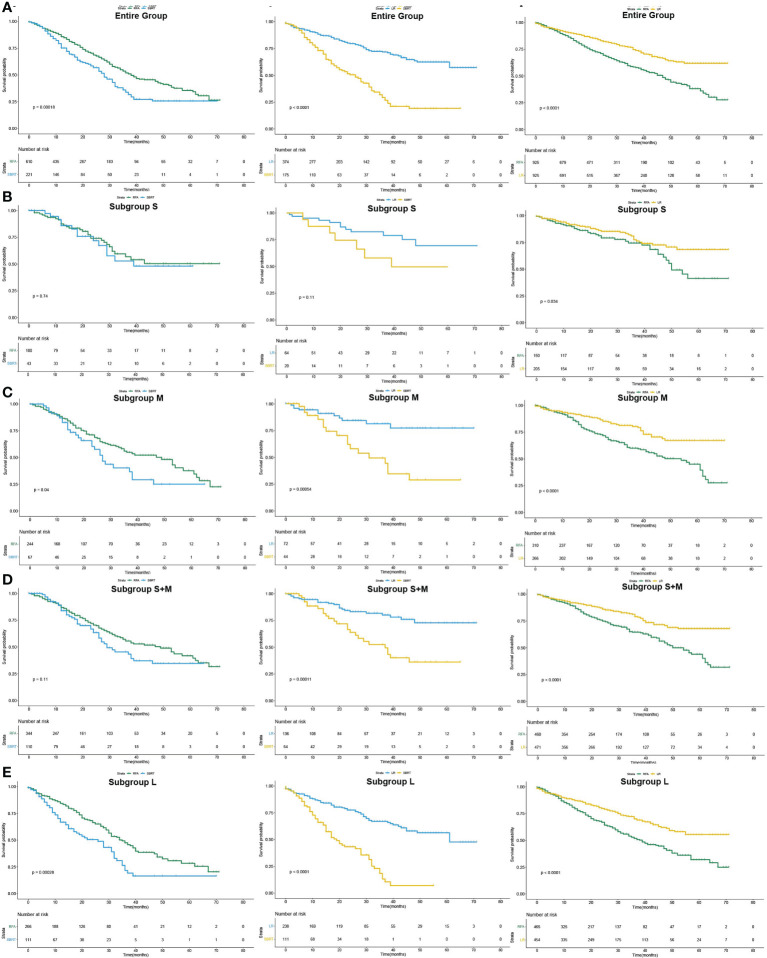
Pairwise comparison of overall survival curves after PSM of patients who underwent SBRT, RFA and LF as initial treatment (Kaplan–Meier method). **(A)** for the entire population, **(B–E)** for subgroup S, M, S+M and L, respectively.

After PSM, the median OS difference between the SBRT group and the LR group or the RFA group in patients of subgroup S was not statistically significant (SBRT, 39 months *vs*. LR, unreached, *p*=0.109; SBRT, 39 months *vs*. RFA, unreached, *p*=0.744), but the median OS of the RFA group was significantly worse than that of the LR group (50 months *vs*. unreached, *p* < 0.001). In the patients of the subgroup M, the median OS of those who took SBRT as the initial treatment was worse than those who had RFA (27 *vs*. 49 months, *p*=0.040), but the median OS difference between the SBRT group and the RFA group in the patients of subgroups S and M was not statistically significant (SBRT, 29 months *vs*. RFA, 49 months, *p*=0.105). Among HCC patients with tumors larger than 2 cm, the median OS of LR patients was the best (unreached for subgroups M and L) compared with either SBRT or RFA group (all *p*<0.001). Detailed results of mOS and HR of each treatment modality are shown in **Table 4**, and survival curves of each group after PSM are shown in **Figure 3**. Compared with either RFA or LR, SBRT showed no significant difference in subgroup S (RFA: hazard ratio, 0.900, *p*=746; LR: hazard ratio, 0.476, *p*=119). For HCCs measuring ≤3 cm (subgroups S and M), SBRT did not significantly differ in the risk of death compared with RFA (HR, 0.749, *p*=0.109), but LR was associated with significant decreased risk of death than SBRT (HR, 0.345, *p*<0.001). In subgroup M, both RFA and LR were associated with lower risk of death compared with SBRT (RFA: hazard ratio, 0.643, *p*=0.043; LR: hazard ratio, 0.484, *p*<0.001, respectively). LR was associated with significantly lower risk of death than SBRT and RFA in group L (HR, 0.284 *vs*. SBRT; HR, 0.557 *vs*. RFA, all *p*<0.001). The detailed results are shown in **Table 5**.

**Table 4:** Results of Kaplan-Meier analyses and median survival time after PSM.

Groups		SBRT vs RFA		SBRT vs LR		RFA vs LR	
		SBRT	RFA	SBRT	LR	RFA	LR
Entire	Median Survival Time, month (95% CI)	28.0 (23.77-32.23)	38.0 (31.80-44.20)	26.0 (19.98-32.02)	–	47.0 (42.80-51.21)	–
	*P* value	< 0.001		< 0.001		< 0.001	
Subgroup S	Median Survival Time, month (95% CI)	39.0 (-)	–	39.0 (-)	–	50.0 (42.88-57.13)	–
	*P* value	0.744		0.109		< 0.001	
Subgroup M	Median Survival Time, month (95% CI)	27.0 (22.53-31.47)	49.0 (35.25-62.75)	31.0 (12.37-49.63)	–	54.0 (44.31-63.09)	–
	*P* value	0.040		0.001		< 0.001	
Subgroup S&M	Median Survival Time, month (95% CI)	29.0 (20.06-37.95)	49.0 (36.71-61.29)	37.0 (25.30-48.70)	–	50.0 (42.96-57.04)	–
	*P* value	0.105		< 0.001		< 0.001	
Subgroup L	Median Survival Time, month (95% CI)	26.0 (17.66-34.34)	35.0 (29.64-40.36)	19.0 (13.92-24.08)	61.0 (-)	39.0 (31.90-46.10)	–
	*P* value	< 0.001		< 0.001		< 0.001	

SBRT, stereotactic body radiation; RFA, radiofrequency ablation; LR, Liver resection;

CI, confidence interval.

**Table 5:** Hazard ratio of treatments in groups with different tumor sizes after PSM.

Groups		SBRT vs RFA		SBRT vs LR		RFA vs LR	
		SBRT	RFA	SBRT	LR	RFA	LR
Entire	Hazard Ratio (95% CI)	1	0.643 (0.51-0.81)	1	0.319 (0.24-0.43)	1	0.542 (0.45-0.65)
	P value	< 0.001		< 0.001		< 0.001	
Subgroup S	Hazard Ratio (95% CI)	1	0.900 (0.48-1.70)	1	0.476 (0.19-1.21)	1	0.617 (0.39-0.97)
	P value	0.746		0.119		< 0.001	
Subgroup M	Hazard Ratio (95% CI)	1	0.643 (0.43-0.99)	1	0.289 (0.14-0.61)	1	0.484 (0.34-0.68)
	P value	0.043		0.001		< 0.001	
Subgroup S&M	Hazard Ratio (95% CI)	1	0.749 (0.53-1.07)	1	0.345 (0.20-0.61)	1	0.519 (0.40-0.68)
	P value	0.109		< 0.001		< 0.001	
Subgroup L	Hazard Ratio (95% CI)	1	0.563 (0.41-0.77)	1	0.284 (0.20-0.41)	1	0.557 (0.44-0.70)
	P value	< 0.001		< 0.001		< 0.001	

SBRT, stereotactic body radiation; RFA, radiofrequency ablation; LR, Liver resection;

CI, confidence interval.

## Discussion

LR is the first option for patients with early-stage HCC besides liver transplantation (3). Although the 5-year survival is promising in patients with very early HCC treated with resection, the high rate of complications is the major limiting factor for hepatectomy (16). RFA can achieve similar LCRs for small HCC compared with LR, but its efficiency decreases obviously in lesions larger than 3 cm (17). SBRT is an emerging alternative intervention in patients infeasible for surgery, but the cutoff of tumor size for its optimal application is still under controversy (11–13). In this study based on large population, we compared the survival of SBRT, RFA, and LR as the initial treatment in patients with AJCC stage I HCC with respect to different tumor sizes. After PSM, LR was associated with better survival than RFA and SBRT in the entire cohort, which was consistent in all subgroups of different tumor sizes. The survival differences among three interventions varied with tumor sizes. In patients with tumor size ≤2 cm, the mOS differences were not statistically significant within the three treatment groups. For patients with tumor size of <3 cm (subgroups S and M together), the difference in OS between SBRT and RF group did not reach statistical significance, while when we look into subgroup M, the mOS of the RFA group was significantly better than that of the SBRT (49 *vs*. 27 months, *p*=0.040). Thus, 3 cm might not be the optimal cutoff for comparing SBRT and RFA, as the different outcomes in patients of subgroup M might be diluted to insignificant level by the similar outcomes in patients of subgroup S.

The findings of our study are highly clinically relevant, since the number of HCC patients diagnosed at AJCC stage I has been increasing nowadays due to pervasive surveillance for population at high risk for HCC, especially HCCs measuring ≤2 cm (3). The patients with HCC are shifting to more elderly population, and age-related comorbidities may become significant contradictions for LR considering treatment tolerability (18). The results of our study implied that SBRT could become a comparable alternative treatment to LR for patients with tumors ≤2 cm, especially the ones who do not tolerate invasive therapy and anesthesia. This might explain the much higher proportion of patients older than 75 years in the group of SBRT (27.2%) compared with the group of RFA (12.8%) and LR (13.8%) in the present study.

Studies have reported that SBRT has higher LCRs than RFA for HCCs measuring >2 cm, but it may not be rigorous enough to take LCRs as a surrogate marker for survival, since disease progression can occur in untreated lesions (11, 19). Berger et al. reported that the OS of SBRT is similar with that of local ablation for HCCs measuring <3 cm, while ablation has better survival benefits for patients with HCCs larger than 3 cm, which is similar with the results in our study (13). However, the sample size of their study was relatively small, although it was multi-institutional, and they included various modalities of ablation such as photodynamic therapy and cryosurgery. RFA is still the standard local ablative modality for HCC and has superior LCRs and survival benefits than other ablative modalities, which makes RFA more widely performed than others, but studies comparing OS of RFA and SBRT are still lacking (20). One study has demonstrated that RFA yields superior survival than SBRT in patients with stage I or II HCC regardless of tumor sizes (12). Among the patients receiving SBRT in their research, 31.76% of them had stage II HCC, suggesting that some of them could have vascular invasion (21). This invasion is associated with abnormal microvasculature, which leads to hypoxia due to poor transportation efficiency (22). Hypoxia is a negative factor for the efficiency of SBRT, since irradiation depends on oxygen for its cytotoxic effects (23). In our study, we only enrolled patients with stage I HCC, and the OS of SBRT did not differ significantly from that of RFA in patients with HCCs ≤2 cm in size, which implied that SBRT might be more feasible for lesions without vascular invasion but not simply depending on tumor sizes. Although the efficiency of SBRT was comparable to RFA for HCCs up to 2 cm in size, cost effectiveness of each modality needs to be considered. One study has reported that SBRT is not as cost effective as RFA for inoperable HCCs; further research is still urged (24). For HCCs measuring >2 cm, RFA provided more survival benefits than SBRT in our study, which was similar with other studies (12, 13).

The survival time of patients with HCC larger than 2 cm and treated with LR was significantly longer than that of those treated with SBRT, while similar OS were achieved by SBRT and LR in HCC patients whose tumor size ≤2 cm in the present study. Few studies have directly compared the survival of patients treated with LR and SBRT. Su et al. reported that there was no significant difference in OS and progression-free survival between patients treated with SBRT and LR for HCC patients whose tumor size ≤5 cm (14). However, the sample size of this retrospective single-institution research was relatively small (n = 82 for SBRT and n = 35 for LR), which might cause biased results. Thus, well-designed clinical trials are demanded to further elucidate whether SBRT could reach comparable survival to LR for HCCs ≤2 cm in size.

This study has several limitations; although the sample size of the entire study population was large, detailed clinical information such as liver function and etiology of HCC was unavailable. The confounding factors between subgroups might not be controlled ideally even with stratified analyses, multivariate Cox regression model, and PSM. The doses of radiation for patients treated with SBRT were also not captured. Thus, our research might not be able to help figure out the optimal protocol for SBRT and might underestimate the value of SBRT in HCC because of the nonstandard usage. Recurrence-free survival could not be obtained, which is a very important outcome to measure the efficacy for different interventions, since salvage treatments after recurrence would affect OS. Furthermore, the SEER database consists of patients only from the United States; whether the present results are applicable in other populations remains uncertain. Large globally randomized controlled trials are needed to verify the value of SBRT in HCC. Finally, the rationale for the study is not completely novel; the authors are looking forward to methodological innovations by future studies to raise evidence whether SBRT improved outcomes for HCC patients.

## Conclusions

For HCC ≤2 cm, SBRT could be used as an alternative treatment for RFA. Liver resection was the best choice for HCC patients with tumors size larger than 2 cm. In patients with HCC lager than 2 cm who are not suitable for hepatectomy, RFA could provide better long-term survival than SBRT.

## Data availability statement

The datasets presented in this study can be found in online repositories. The names of the repository/repositories and accession number(s) can be found in the article/supplementary material.

## Author contributions

Z-LY and LX designed the study. X-QS, Y-HT, P-YX collected and interpreted the data. Z-LY and LX prepared the final draft and all authors were involved in the initial drafting, review, and approval of the manuscript and the decision to submit it for publication. All authors contributed to the article and approved the submitted version.

## Funding

This work was funded by the National Key R&D Program of China (2020YFE0202200) and the National Natural Science Foundation of China (81772589).

## Conflict of interest

The authors declare that the research was conducted in the absence of any commercial or financial relationships that could be construed as a potential conflict of interest.

## Publisher’s note

All claims expressed in this article are solely those of the authors and do not necessarily represent those of their affiliated organizations, or those of the publisher, the editors and the reviewers. Any product that may be evaluated in this article, or claim that may be made by its manufacturer, is not guaranteed or endorsed by the publisher.

## Abbreviations

SBRT, stereotactic body radiation; RFA, radiofrequency ablation; LR, Liver resection; PSM, propensity score matching; AFP, alpha-fetoprotein; CI, confidence interval; HR, hazard ratio; IQR, interquartile range; K-M, Kaplan–Meier; OS, overall survival; mOS, median overall survival; LCR, local control rate; RCT, randomized controlled trial; US, United States; AJCC, American Joint Committee on Cancer; SEER, Surveillance, Epidemiology, and End Results database.
